# Editorial

**Published:** 2007-07

**Authors:** AJ Brink

**Affiliations:** Editor, Cardiovascular Journal of Africa

This issue of the Cardiovascular Journal of Africa is a contribution to the Council of Science Editors’ global theme issue on poverty and human development.

The poverty-stricken nations of the world lie largely between latitudes 15° south and 30° north of the equator, in particular in tropical and subtropical areas. Endemic diseases are rife in these regions, some of which are malaria, schistisomiasis, helminthiasis, hydatid disease, amoebic dysentery, spirochaetosis and rickettsia. Other diseases of uncertain origin are also common, such as cardiomyopathies.

An in-depth study on the annual per capita income of countries in relation to their environmental temperature ranges, with its implications for global warming, confirms^1^ that the poorer nations are in the warm tropical and subtropical climates. Another article clearly illustrates that poverty is largely entrenched in areas with a tropical climate and lack of access to the sea.^2^

Disease is considered to be a causative factor of a nation’s poverty but low productivity, lack of education and other factors are also thought to play a role. However, it is possible that disease is also the historical background for all the other suggested factors leading to the impoverishment of those living in tropical and subtropical climates.

In these regions, disease has resulted in poor physical and mental development of the people. Sickness has weakened them, making them subjects of slavery and exploitation for cheap labour, and foreign countries have stripped them of their natural resources. The industrial revolution passed them by and the modern levels of unprecedented technological development could not be acquired due to lack of education and opportunity. A constant vicious circle of physical and educational backlogs leading to an inability to maintain health and compete for survival with the more affluent nations has left sub-Saharan Africa in dire straits.

The other major adverse influence on these populations occurred in those who were in transition from a rural setting to more developed city environments. A cultural shock awaited the underprivileged rural populations in transition when they came face to face with new life styles while trying to resettle in the adopted community. They increasingly became victims of hypertension, coronary artery disease and the emerging metabolic syndrome comprising obesity, hypertension and diabetes.

In this issue we draw attention to some of these cardiovascular situations and conditions, which may either be the consequence of, or at the very least associated with poverty and the adaptation of persons in transition from one culture to another. Conditions such as hypertension, the pattern of heart failure, the prevalence of chronic heart disease, and perspectives on malnutrition and underdevelopment related to heart disease are described in the articles. Vascular risk factors relating to stroke and coronary artery disease, including data on cut-off points for abdominal obesity have been studied and are presented. What is described in specific African states applies in general throughout the subcontinent.

Only large-scale educational drives and the eradication of disease can bring a fertile subcontinent with abundant natural resources to a point where it can be self-sufficient and develop the people to their full potential. These programmes of education and the reduction or eradication of disease in the impoverished world will have to be engaged in rapidly if there is to be any hope of achieving healthy lives with average life expectancy in these populations.
